# Value judgment of health interventions from different perspectives: arguments and criteria

**DOI:** 10.1186/s12962-018-0099-6

**Published:** 2018-04-17

**Authors:** Karin M. Vermeulen, Paul F. M. Krabbe

**Affiliations:** Department of Epidemiology, University of Groningen, University Medical Center Groningen, P.O. Box 30.001, 9700 RB Groningen, The Netherlands

**Keywords:** Judgment, Arguments, Criteria, Decision-making, Health policy

## Abstract

**Background:**

The healthcare sector is evolving while life expectancy is increasing. These trends put greater pressure on healthcare resources, prompt healthcare reforms, and demand transparent arguments and criteria to assess the overall value of health interventions. There is no consensus on the core criteria by which to value and prioritize interventions, and individual stakeholders might value specific elements differently. The present study is based on a literature review that retrieved the most widely recognized arguments and criteria used in decision-making. The aim was to compile a smaller set of arguments and criteria that would seem most relevant to different stakeholders.

**Methods:**

A literature review was performed in Medline and EMBASE. The initial search retrieved over 2000 articles and documents of relevant committees. A selection was made based on their reference to healthcare, policy issues, or social justice. Finally, 84 papers were included. Data extraction took place after appraisal of the articles. A full table was made, including all arguments and criteria found; next, identical or largely overlapping arguments were excluded. The remaining arguments and criteria were assessed for relevance and a reduced set was compiled.

**Results:**

The final set included 25 arguments and criteria, categorized by type (clinical, social justice, ethical, and policy). For each argument and criterion, relevance to stakeholders was scored on three levels (not, partly, and completely relevant).

**Conclusions:**

Many arguments and criteria play a role in making value judgments on health interventions, but not all are relevant to all interventions. Moreover, they may interact with each other. A viable way to deal with interacting and possibly conflicting arguments and criteria might be to arrange public discussions that would evoke different stakeholders’ perspectives.

## Background

Due to ongoing advances in medicine and health technology and the broadening scope of health services, health systems face the challenge of prioritizing new technologies [1, 2]. Furthermore, as life expectancy increases, the proportion of the elderly will continue to grow, though they may not age in good health [3, 4]. The additional burden these trends place on healthcare resources, explains why many national health systems have recently introduced reforms [5]. All assessments performed for healthcare systems and all reimbursement decisions for new health technologies depend on an assessment of benefits or value [6, 7]. To ascertain this value requires an explicit framework defining the goals of a health system against which outcomes can be judged and performance quantified [8].

In the UK, the National Institute for health and Clinical Excellence (NICE) has four guidance programs. ‘Technology appraisals’ and ‘Clinical guidelines’ take the clinical effectiveness and cost-effectiveness of an intervention into account. Under the program ‘Interventional procedures’, clinical efficacy and safety of the intervention are taken into account, whereas cost-effectiveness is not. ‘Public health’ takes effectiveness and cost-effectiveness of public health activities into account [9, 10].

In the Netherlands, a more elaborate framework is applied. The Minister of Health, following advice from governmental organizations (e.g., National Healthcare Institute (Zorginstituut Nederland; formerly called Health Insurance Board, CVZ), decides which interventions are to be covered by health insurance. Since the 1990s, the so-called Dunning criteria have been used to formulate this advice [11]. Currently, the four principal criteria are need, effectiveness, cost-effectiveness, and feasibility [12, 13].

According to the Belgian Drug Reimbursement Committee, an application for reimbursement is needed for Class 1 drugs, i.e. those with more therapeutic value than the existing alternatives. The decision on reimbursement is based on multiple factors, including the therapeutic value of the drug, its price, its importance in clinical practice, its budget impact, and cost-effectiveness [14].

On a global level, the World Health Organization (WHO) mentions three ethical principles for prioritizing: efficiency (maximizing population health), fairness (minimizing health differences), and utility (greatest good for the greatest number) [15].

Many national and individual studies have examined the methods and principles underpinning the assessment of health interventions [6, 16–20]. Overall, results of these studies suggest heterogeneity of the identified criteria [17], convergence among decision-makers on the relevance of criteria [20], and divergent operationalization of (the same) criteria [17]. It has been suggested that some of the discrepancies found are strongly related to contextual factors [20]. Thus, there is no consensus on the core criteria by which to value health interventions, and some authors even assert that the fundamental principles are poorly defined [21–23]. According to Golan [2], the key to evaluating the success of prioritization efforts lies in articulating appropriate principles as well as formulating reasons that are grounded in clear value choices [24].

Apart from these fundamental concerns, the valuation of interventions and the relevance of criteria might differ depending on who is the ‘judge’ (clinicians, health authorities, general population, or patients). Different stakeholders might value different determinants that could affect coverage decisions in different ways. In addition, the growing involvement of patients in healthcare is a factor in policy-making.

The aim of the present study is to identify the most widely recognized arguments and criteria that are used in making decisions about patient treatments or in prioritizing health interventions. Arguments and criteria are hard to define. Some elements can be clearly regarded as criteria, and others as arguments, but there is also quite some overlap between the two. Criteria are considered here as a rule or principle that has to be met or a standard by which one judges, whereas an argument refers to a way of reasoning (group of statements) to be in favor for something or not. We intend to identify and summarize the most salient ones, to inform a broad audience of policy makers and health care professionals.

## Methods

### Search strategy

An electronic search was performed in the Medline and EMBASE databases (February 2018). Since our aim was to identify and summarize the most salient arguments and criteria, to inform a broad audience, a review of the literature was carried out, instead of a systematic review. A literature review can cover a wide range of subjects at different levels of completeness and comprehensiveness. [NCBI] Search terms were related to priority-setting, decision-making, decision tools, and resource allocation. Logical combinations were made with the following perspectives: patient, healthcare professional, clinician, decision-maker, health authorities, and general public/population. After the initial search, further refinements were made in light of expert opinion (KV, PK).

The initial search retrieved over 2000 articles, of which a selection was made on the basis of their reference to healthcare, policy issues, or social justice. The references in these articles were searched and additional papers (about 10) were included. Then five policy documents relating to social value judgment and economic evaluation methodology were found on websites and added to the set. Ultimately, 84 documents were included in the review.

### Procedure

After performing the search, the articles were examined to discern which arguments and criteria they concerned. A table was made containing all of the arguments and criteria mentioned. The next step was to exclude identical and largely overlapping arguments. Finally, using the same procedure, the remaining arguments and criteria were assessed for relevance.

## Results

After retrieval of all the different arguments and criteria that were used or put forward in the identified literature, we observed that most of these can be assigned to four broad categories: clinical, social justice, ethical, and policy. ‘Clinical’ denotes all those issues that arise in daily practice when medical service is offered to patients. ‘Social justice’ (or distributive justice) concerns issues raised outside medical practice, namely in the interdisciplinary field of political science and political philosophy. The term generally refers to a set of institutions that enable people to lead a fulfilling life and be active contributors to their community. Such arrangements fall under entities dealing with (international) law, public services, labor rights, regulation of markets, and healthcare. Pursuit of social justice is laudable, but medical doctors, academics, and patients face the sobering reality of limited resources and the need to devote tax revenues to other important sectors (e.g., education, military, social security). Ethical issues are mainly commented on by clinicians, politicians, journalists, and philosophers, and supervised by the latter. Arguments raised in this discourse mainly concern individuals and less groups of patients (i.e., social justice, priority setting). Many are specific and have a narrow focus (e.g., end-of-life decisions, dignity). Most politicians have a political view that comprises specific considerations related to social justice and ethical issues, which have to be molded into a ‘compromise’ for finding effective support from other politicians and the public. Overall, policy issues are aligned with efforts to keep things manageable and under control, while the clinicians are mainly concerned with doing their best for their patients.

**Fig. 1:**
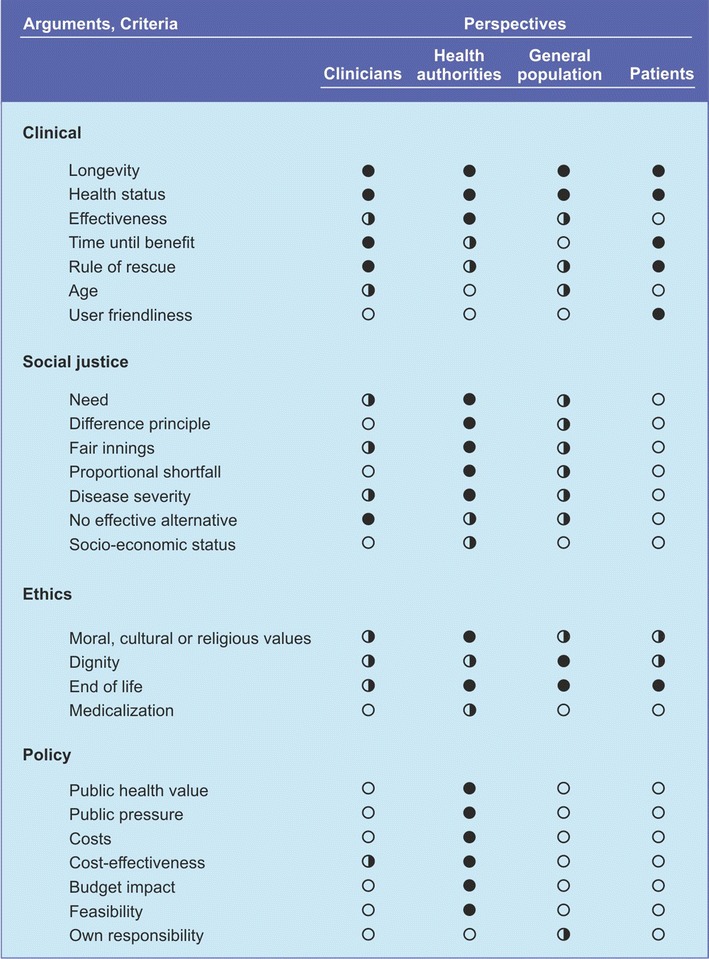
Arguments and criteria for resource allocation categorized into four domains and indications of the relevance of these arguments and criteria to different stakeholders (open circle = not important, half open circle = some importance, closed circle = important)

Some of the arguments and criteria can play a role in multiple categories. For example, ‘Rule of Rescue can be regarded as a clinical issue, but it can also be seen as an ethical aspect. Therefore, our schematic representation should not be regarded as absolute (Fig. 1).

### Clinical issues

Clinical issues, health benefits, or health outcomes traditionally play an important role in many healthcare decision-making procedures. While the terminology varies widely, the decision criteria of most healthcare systems explicitly include the health outcomes and benefits of an intervention. Some arguments refer to these directly; others mention the health status or demographic characteristics of the individual or size of the targeted population [25]; and one argument can also be seen as a principle (Rule of Rescue) [23, 26].

#### Longevity

Longevity can be seen as a characteristic of health gain [25]. ‘Life-saving’ or life-extending procedures are highly valued, significantly more highly than care interventions. There is a heroic element in the act of life-saving, and it has traditionally been one of the main things expected of physicians (see also: Rule of Rescue).

#### Health status

The assessment of medical interventions and healthcare services also takes ‘health status’ or ‘quality of life’ into account. These measures are relevant because, ultimately, the goal of all health interventions is to improve the patient’s perceived health condition. Health status reflects an individual’s relative level of wellness and illness, taking into account the presence of biological or physiological dysfunction, symptoms, and functional impairment. Most measures of health status include key indicators such as physical function, sensation, self-care, cognition, pain, and discomfort [27].

#### Effectiveness

The criteria mentioned under the heading of ‘effectiveness’ or clinical benefit include the following: general benefits, effect on mortality, efficacy, safety, adverse effects, adherence, effect on longevity, and effect on health status (i.e., quality of life), and number in need of treatment. In that sense, effectiveness captures several of the distinct criteria that are classified here as ‘clinical’. *Individual* health benefits strongly influence policy decisions in various countries [28]. The number of patients in ‘need of treatment’ adds information to the decision about the likelihood that a *population* will benefit from a medication or intervention [29, 30].

#### Time until benefit

‘Time until benefit’ is tied to the judgment of whether a patient’s life expectancy is long enough to benefit from the intervention. Medication for symptom relief, such as analgetics, may show benefits in a short time and would continue to benefit all patients, including those close to death. Medications used for prevention may have a time until benefit of years. Unlike the number needed to treat, which adds information about the population that will benefit, the notion of time until benefit may be useful in discussions on individual patients as well [29]. From a patient’s perspective, the question ‘when will it help?’ is often just as important as the question ‘how much will it help?’ From a professional’s viewpoint, looking at the balance between an older patient’s life expectancy and the lag in time to benefit might help clinicians identify which patients are more likely to be helped and which ones are more likely to be harmed by the intervention [31].

#### Rule of Rescue

Tension sometimes arises between the injunction to do as much good as possible, and the injunction to rescue identifiable individuals in immediate peril, regardless of costs. This is called the ‘Rule of Rescue’ [23, 26]. The principle was named by Jonsen in 1986 [32] in a paper describing our practical incompetence to deal with life-saving or life-sustaining technologies. Our moral response to the imminence of death demands that we rescue the doomed [33]. As such, the Rule of Rescue can also be seen as an Kantian ethical imperative and inherent ethical phenomenon. One of the most conspicuous features of this rule, according to McKie, is the tendency to disregard opportunity costs (benefits or values of something that must be given up to acquire or achieve something else) when an identifiable individual is visibly threatened [34]. There is a tendency to act first and consider the costs later.

#### Age

In many countries there is a strict (ethical) limitation to considering age as a criterion in decision-making on non-preventive medical treatments. Preventive health care aims to delay the onset of illness and disease and to prevent untimely and premature deaths [35]. In many guidelines, age is the main criterion for recommending preventive interventions. Nevertheless, in recent years concerns about equity of access to treatments have focused on ageism. As a result, preventive interventions are encouraged regardless of age, although this can be harmful to the patient and expensive for the health service. Yet in many situations, age (or life expectancy) can be a good predictor of success [31]. Also, there is increasing awareness that at a certain age, when frailty becomes prominent, many medical interventions lose their effectiveness and may even be considered inappropriate.

#### User-friendliness

‘User-friendliness’ (convenience) is often seen as an aspect of therapeutic value, as it may result into better adherence in the case of drugs. Thus, relevant for both patient in terms of clinical outcomes, as well as for the prescriber, this issue is not often mentioned as a criterion by decision makers.

### Social justice

‘Social justice’ is the ability to realize one’s potential in the society one lives in. It generally refers to a set of institutions that enable people to lead a fulfilling life and be active contributors to their community. Thus, its goal amounts to human development, and the relevant institutions are usually taken to include education, healthcare, social security, labor rights, as well as a broader system of public services, progressive taxation, and regulation of markets. All these constituents are obligated to ensure a fair distribution of wealth, equality of opportunity, and no gross inequality of outcome [35, 36]. Social justice is closely related to distributive justice; the latter means ensuring that individuals have both fulfilled their societal roles and received what was due to them from society. Distributive justice concerns the nature of a socially just allocation of goods (e.g., medical treatments) in a society. This subject has been given considerable attention in philosophy and the social sciences (e.g., psychology, economics) [37]. Inextricably connected to social justice are themes of values and ethics (see: below).

#### Need

‘Need’ reflects an impairment relative to a ‘normal’ health status. The term must also incorporate a patient’s capacity to benefit from treatment [21, 38], usually expressed as the effectiveness or appropriateness of an intervention [2, 38]. Healthcare systems include need-based principles, such as the severity of the condition (the Netherlands, Sweden, Norway, Denmark, and France) [2], the degree of clinical need (NICE) [9], or the importance of human disabilities (Australia) [39]. As such, one of the key principles of justice raised in academic discussions (alongside maximizing and egalitarian principles, which are utilized in the field of economics [40]) is need.

#### Rawls’ difference principle

**Fig. 2:**
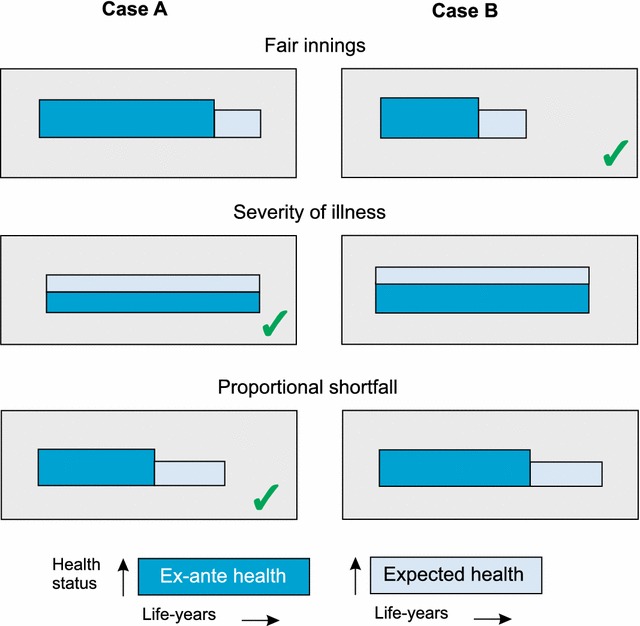
Schematic representation of three principal distributive mechanisms as part of social justice considerations (areas are representing health status and life years)

A well-known theory of social justice forms the core of a work on political philosophy and ethics by John Rawls. Originally published in 1971 [41], it was revised in 1975 and again in 1999. Rawls attempted to solve the problem of distributive justice. He analyzed the principles of justice that would be adopted by rational self-interested people whose ‘initial situation’ is characterized by a ‘veil of ignorance’ that conceals all factors relevant to the determination of their individual fortunes in society [42]. The second of these is the ‘difference principle’. Policies and structures are to be arranged to diminish social and economic (hence: health) inequalities and benefit the least-advantaged members of society. Although his work is one of the cornerstones of political science, other more detailed arguments have been raised dealing with social justice in the context of health (Fig. 2).

#### Fair innings

The ‘fair innings’ approach centers on the feeling that everybody in Western countries is entitled to a ‘normal’ number of life years. This argument is also part of the disability-adjusted life year (DALY) approach used by the WHO [43]. The concept of fair innings was introduced in the field of health economics and political philosophy by the British economist Alan Williams, although it grew from earlier roots [44, 45]. Williams proposed that life years gained by people facing less than fair innings should be valued more highly than life years gained by people expecting to have fair innings or better.

#### Proportional shortfall

‘Proportional shortfall’ poses that priority should be determined by the proportion of quality-adjusted life years (QALY) that people lose relative to their remaining life expectancy due to some illness. Proportional shortfall compares individuals in relative terms to determine who is worse off [46]. Thus, this equity concept combines elements of the two principles mentioned above. Empirical studies based on hypothetical scenarios found that people’s preferences were most in line with the fair innings arguments, followed by the proportional shortfall principle, and then the severity-of-illness or disease severity criterion (see below) [47, 48].

#### Disease severity

In many European countries, such as Norway, Sweden, France, Germany, and Spain, reimbursement decisions are informed by considerations of severity [49]. Society’s appreciation of medical interventions rises sharply with the increasing severity of the patients’ condition or health status. This criterion is often referred to as a ‘concern for the worse-off’ or ‘disease severity’. The severity approach is drawn from a number of well-known theories of distributive justice asserting that the worst-off in society have special and legitimate claims [41, 49–51]. It is, however, not always clear what is meant by severity, and who the worst-off are. We may try to rank patients that are worse-off with regard to their health status and relate this ranking to how much they are helped by treatment [52]. Yet there are different ways of approaching this. First, one can look at current health status and expected improvement. From this angle, an improvement has more value if the current health status is worse but the expected improvements are the same [53]. A second approach also considers the patient’s past health status.

#### No effective alternative

When a new beneficial treatment is introduced for which treatment was previously unavailable, the standard efficacy and cost-effectiveness thresholds are often leveraged in order to give this class of patients at least the opportunity to relieve their health condition and to avoid blocking innovation of potential treatment options in this area. A number of papers report on the preferences for availability of alternative treatment and all found that a disease with no alternative treatment was given priority [54–56]. More or less the same train of thought can be observed for so-called orphan drugs. Pharmaceutical companies develop these for rare diseases and often have no interest in producing them from a business perspective. Despite not qualifying as cost-effective, many orphan drugs are subsidized in the EU member states [57]. The rationale for less strict reimbursement thresholds is that the limited production of these drugs justifies their high price. Another argument is that very expensive new drugs or treatments should not be withheld from patients if no effective alternative treatment is available.

#### Socio-economic status

Seldom is ‘socio-economic status’ mentioned as an independent criterion for prioritization. It relates to the principle of fair innings, which assumes that patients are entitled to their expected number of life years [44, 45, 58]. It is also related to life expectancy, in that patients with a lower socio-economic status are less likely to achieve their ‘fair innings’ than patients with a higher one. Thus, programs that generate health effects predominantly in patients with low socio-economic status would be preferable on the grounds of equity considerations [59].

### Ethics

Moral issues embrace various ethical concepts such as human dignity, religious or cultural values and convictions, but also the question whether a new medical intervention is subject to criticism for medicalization, overdiagnosis, or overtreatment. Medical ethics (or bioethics, health ethics) emphasis the rights of individuals, often the doctor-patient relationship. Less attention is given to the institutional and societal implications [60]. Many of these more population based moral considerations are addressed in the context of policy science, political philosophy, economics and other special disciplines, but can be classified as moral too (see: Social justice).

#### Moral, cultural, and religious values

Hofmann [61] provides an apt overview of morally relevant questions with respect to assessing health technology. Moral challenges may be posed by the involvement of third parties such as (organ) donors, bio-bank contributors, proxies, surrogacy (e.g., donation), and family members (in the case of genetic diagnostic testing) [61].

Practices that may challenge ‘cultural or religious convictions’ are, for example, blood transfusions, which Jehovah’s Witnesses refuse to accept, and vaccination, which is unacceptable to the (Dutch) orthodox protestant community [62]. Reimbursement decisions related to such interventions are valued differently by different groups, based on their spiritual beliefs. Niezen [63] claims that in dealing with the abstract reports on cost-effectiveness and need (severity of illness), the Dutch committee for appraising medicines ACP puts the person who is ill at the center of the appraisal.

#### Dignity

Care systems, traditions, and philosophies have arisen because individual human beings have been considered so valuable that they cannot just be left at the mercy of their sickness and suffering [64]. Dignity becomes particularly relevant when medicine has less to offer to patients or when people become very old and fragile. In that case, the emphasis shifts from cure to care. Especially for elderly, handicapped, and mentally disabled people, it is the tradition of ‘care’ that matters most.

#### End of life

‘End of life’ care, in its narrow sense, is healthcare for patients in the final hours or days of their lives. However, the concept also applies more broadly, covering care of those with terminal illness that is advanced, progressive, and incurable. Management of symptoms plays an important part in this type of care. Regarding decision-making on coverage, a prime example of dilemmas relating to end of life is cancer care. For advanced cancer, difficult trade-offs must be made between the limited health benefits of the drugs and their high cost [65].

#### Medicalization

‘Medicalization’ is the process whereby social issues, or issues related to well-being, are looked at from a medical point of view. Development of new tests and medicines can increase the risk of medicalization or change the perception of disease. For example, IVF turned a social matter (childlessness) into a medical one (infertility) [61].

### Policy objectives

Governments, health authorities, and other formal institutions consider a variety of policies regarding national health systems. Of course, these institutions also consider the overall values that were discussed above. Apart from these there are numerous criteria that policy-makers typically deal with.

#### Public health value

In many European countries, the two main objectives of publicly funded healthcare are to maximize population health and to reduce inequality in health across groups within the population. Both objectives are subject to resource constraints and together they constitute ‘public health value’. A socio-political target such as well-being and a favorable economic condition such as high productivity are more easily attained in a nation of healthy people. Part of the public health value consists of medical interventions that are considered valuable, not so much for the individual but for society at large (e.g., infectious diseases).

#### Public pressure

In an Australian study, decision-makers were asked to identify factors that influence resource allocation in healthcare [66]. Political factors were mentioned most frequently. Among others, one important element of these political factors was the climate of opinion in society and pressure groups. The effect of pressure groups or ‘public pressure’ in general may have great impact on actual healthcare allocation decisions [67].

#### Costs

‘Costs’ of healthcare rose rapidly after the Second World War in all developed countries. Many reasons can be put forward to explain this process: more technical options (e.g., CT scan, transplantation); expansion of the content of healthcare; side-effect of the merits of modern healthcare (e.g., people live longer without fatal diseases but need more care); professionalization. In combination, these factors are responsible for a substantial proportion of government spending. This has caused the need for policy makers to weigh up the costs to the health system of funding an intervention balanced with the effect [68].

#### Cost-effectiveness

‘Cost-effectiveness’ is an important criterion in many countries [28, 68–71]. It is also called a ‘higher order criterion’ since it is a composite measure of costs and effects. The effects are often expressed in terms of quality-adjusted life years (QALYs). A QALY combines (expected) survival with (expected) level of health status in a single metric [72]. In the Netherlands, cost-effectiveness is an important criterion but no absolute threshold is applied. Only the legitimacy of the cost-effectiveness ratio is assessed [73]. Strict thresholds are rarely set or applied in other healthcare systems [17]. For the last two decades, the ratio of $50,000 per QALY gained has played an enigmatic role as a benchmark for the value of care [74]. It seems that certain factors may lead to higher acceptable costs per QALY gained, for example, in the case of rare diseases (see also: no effective treatment). Alglucosidase alfa (Myozyme), for example, a drug used in the treatment of Pompe’s disease [75], was only accepted for reimbursement in the Netherlands after public discussion and price negotiations with the manufacturers.

#### Budgetary impact

Budgetary impact analysis estimates the financial consequences of adoption and diffusion of a new drug or intervention in a specific healthcare setting such as a hospital or clinic or by a private or public insurer [76]. In particular, such analyses predict how a change in the mix of drugs and other therapies will impact spending on that condition. The reference point for this analysis is the current mix of interventions for the population in question. For instance, reading glasses were not accepted for reimbursement at a certain stage in the appraisal process due to the high budgetary impact of this health aid [13].

#### Feasibility

One of the criteria used to appraise a new intervention is ‘feasibility’ [77]. This criterion is considered a contextual factor and can be evaluated in terms of the available means, such as budget, administrative burden, staff and ward availability, and expertise. Feasibility is also weighed in light of establishing a precedent and other unintended effects, but also in view of laws and legislation.

#### Own responsibility

The overall conception is that each individual is presumed to be responsible for some aspects of his or her health status. Medical doctors are offering their patients the best available treatment, taking no consideration whether the cause of the injury or disease is related to unhealthy lifestyle factors or not. Such lifestyle factors related to unhealthy lifestyle are eating habits, smoking, and even performing extreme sports or excessive sunbathing [78]. In most health care systems unhealthy lifestyle factors are not entered in priority setting. In general, the question of own responsibility is a sensitive one and has a prominent place in ethics (autonomy). A public debate could promote acceptance of decisions on resource allocation in which a person’s ´own responsibility´ and lifestyle factors may have an impact [79]. Objections to public involvement in the discussion are based partly on the assumption that individuals are both subjective and inadequately informed and therefore cannot represent the interests of others [79]. But as a German study demonstrates, engaging in behavior that is harmful to one’s health is generally accepted as a criterion for prioritizing patients, mostly regardless of self-interest [78].

## Discussion

The aim of the present study was to retrieve the most generally recognized arguments and criteria that are used in healthcare decisions. We got the impression that values such as those expressed in social justice arguments are the leading principles. Although subjective, we have attempted to express the level of relevance of the various arguments and criteria by four groups: clinicians, health authorities, the general population, and patients.

First of all, there are obvious differences between the four groups. Clinicians and other health professionals predominantly apply clinical arguments in their treatment decisions. Health authorities do take great interest in policy arguments and also incorporate social justice arguments and clinical criteria in their deliberations. For the general population, the picture is less clear. Apart from some key criteria such as longevity, health status, and ethical concerns for the elderly (dignity, end of life), none of the four broad categories: (clinical, social justice, ethical, and policy) seems to predominate. Among the general population we also observe a moderate interest in social justice criteria. Finally, patients are fairly comparable to the general population, though they seem less interested in social justice arguments.

We identified almost 30 arguments and criteria that are relevant and current. Many others were observed but not mentioned in our overview, because we regard them as either very specific, different names for another concept, or overlapping with another argument or criterion. Examples of such redundancies are the following: timely deliveries, dosages available, years on market, producer’s reputation, and constitutional factor.

Different stakeholders have different interests and therefore use different frameworks in their reflections. The perspective of clinicians is to bring the best care to individual patients. Social justice arguments are deemed relevant from every perspective except that of the patients. This exception bespeaks the human condition: if we are healthy, we may give due regard to meta-conceptions and assign importance to distributive justice and solidarity principles; if we are seriously ill, we are mainly interested in our own condition. Costs are relevant only to policy-makers, which explains why it is up to them to make unpopular decisions.

Enhancing the health status experienced by those who are already quite healthy is a legitimate policy objective. However, many countries pursue a much stronger policy goal of reducing the suffering of severely ill people. This emphasis is largely based on the idea that patients in bad health are in greater need. As such, need is one of the guiding principles of justice that are raised in academic discussions, along with maximizing population health and egalitarian principles that play a greater role in the area of economics and public health [23, 80, 81]. A well-known example of combining different principles in practice has been explored by Hadorn [82] in a study of the Oregon Health Services Commission, which drew up a package of healthcare services that should qualify for public funding. This procedure gives some weight to maximizing health (because relatively ineffective healthcare is not funded) and some weight to distributing in proportion to capacity to benefit (because relatively effective healthcare is funded even if it is not cost-effective).

Various procedures and principles have been put forward to deal in a structured manner with the imperative to prioritize health interventions or programs. Most of these procedures and principles are rather academic. A few countries have a clear policy framework by which to evaluate (mostly new) health interventions. In the UK, under NICE, new drugs have been rejected for reimbursement. In the Netherlands, several drugs that are hardly cost-effective are nonetheless prescribed and reimbursed for other reasons.

In a survey of attitudes represented in the Swedish formulary committees, Anell [83] discerned the five most important criteria for the establishment of clinical guidelines. Among these were three effectiveness parameters: therapeutic effects, adverse effect profile, and probability of patient adherence (e.g., drugs, rehabilitation, lifestyle). Another study examined the current health policy of about 40 countries and observed five criteria: effectivity, safety, level of evidence, severity of disease, and budgetary impact [20]. Safety is often mentioned as a separate criterion for evaluating health interventions, but in fact in most cases, formal safety and quality checks have been conducted for drugs and devices before they are certified to enter the market (e.g., European Medicine Agency). Similarly, ‘level of evidence’ is neither an argument nor a criterion but a precondition.

## Conclusion

Many arguments and criteria seem to play a role in value judgments regarding health interventions. As they may interact which each other, we are dealing with a complex system of different and even conflicting arguments and criteria. It seems that there is no practical, structured way to deal with these factors such that the results can rationally be applied to reimbursement decisions. Some methodological frameworks (e.g., multi-criteria decision analysis) are possible candidates for approaching this complex research question [84]. However, the number of optional criteria is very large, and we lack a limited set of criteria that take precedence under all conditions. An analytical framework based on axiomatic theory or mathematical models, such as multi-criteria decision making, would probably only serve a crude role in prioritizing some distinct healthcare interventions. In the end, elaborate public discussion may be the most viable process facilitating societal consensus about health policy arguments and criteria.
